# The Use of Narrow Diameter Implants in the Molar Area

**DOI:** 10.1155/2016/8253090

**Published:** 2016-05-11

**Authors:** M. Saad, A. Assaf, E. Gerges

**Affiliations:** ^1^Private Practice Limited to Periodontology and Implantology, Tyre 00961, Lebanon; ^2^Unit of Biomaterials and Technology, School of Dentistry, Lebanese University, Beirut 00961, Lebanon; ^3^School of Dentistry, Beirut Arab University, Beirut 00961, Lebanon; ^4^Department of Prosthodontics, School of Dentistry, Lebanese University, Beirut 00961, Lebanon

## Abstract

Implant rehabilitations in the posterior jaw are influenced by many factors such as the condition of the remaining teeth, the force factors related to the patient, the quality of the bone, the maintenance of the hygiene, the limited bone height, the type and extent of edentulism, and the nature of the opposing arch. The gold standard is to place a regular diameter implant (>3.7 mm) or a wide one to replace every missing molar. Unfortunately, due to horizontal bone resorption, this option is not possible without lateral bone augmentation. In this situation, narrow diameter implant (NDI < 3.5 mm) could be the alternative to lateral bone augmentation procedures. This paper presents a clinical study where NDIs were used for the replacement of missing molars. They were followed up to 11 years. Special considerations were observed and many parameters were evaluated. NDI could be used to replace missing molar in case of moderate horizontal bone resorption if strict guidelines are respected. Yet, future controlled prospective clinical trials are required to admit their use as scientific evidence.

## 1. Introduction

There are different definitions for the narrow diameter implant (NDI), starting from small body implant, implant with a reduced endosseous diameter, and narrow body implant to reduced diameter implant. The diameter is always less than or equal to 3.5 mm. Originally, its use was reserved for the replacement of teeth with narrow clinical crowns and/or for limited interdental or interimplant spaces such as in the upper lateral or lower incisors areas [1]. As the observed success rate is similar to that of standard diameter implants (SDIs) [2, 3], it is suggested that implant success is not related to implant diameter. Bone loss around narrow implants was within the same limits as those reported around standard diameter implant [4, 5].

However, NDI finds another indication for its use, namely, with thin ridges. Indeed, following tooth loss, bone collapses in a three-dimensional pattern. The horizontal deficiency or width loss develops in a larger extent [6].

Here, the clinician has two options, either to perform horizontal ridge reconstruction procedures (guided bone regeneration, ridge splitting, and onlay bone graft) or to place an NDI in case of moderate horizontal bone loss [7].

Guided bone regeneration (GBR) is one of the most documented procedures for horizontal bone augmentation in implant dentistry. Clinical and histological aspects are well established [8–11]. However, despite the wealth of documentation, it remains a sensitive procedure as it depends on many factors such as the selection of the appropriate bone substitutes, membrane nature (collagen native, cross-linked, resorbable, nonresorbable, etc.) and membrane fixation screws. Moreover, the need for periodontal plastic surgery for the reestablishment of keratinized tissue is not rare after GBR procedures [12, 13].

The other suitable solution to avoid invasive ridge management techniques in cases of limited ridge width is the use of NDIs [2, 7], therefore broadening their indications. However, it has been avoided in the posterior jaw for prosthetic and biomechanical considerations. The emergence profile of posterior teeth is rarely harmonious with a narrow implant neck. Complications are expected to exceed those generally observed for the standard diameter implant such as implant fracture, abutment fracture, screw loosening or fracture, and ceramic fracture [14–17].

**Figure 1:**
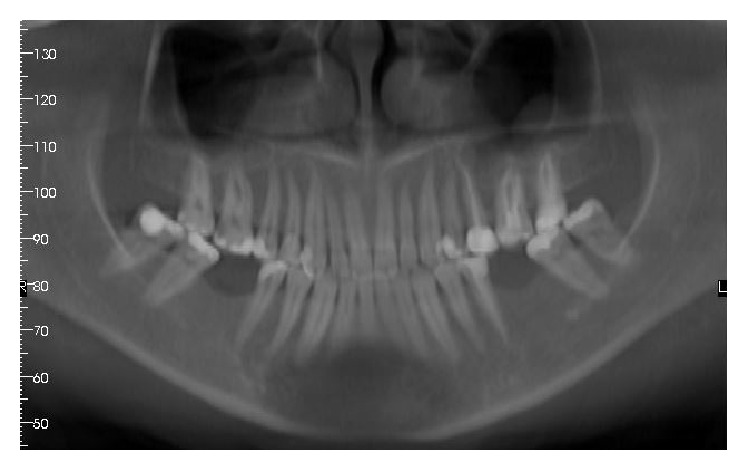
Panoramic X-ray showing teeth loss on the lower 1st molar area.

**Figure 2 fig2:**
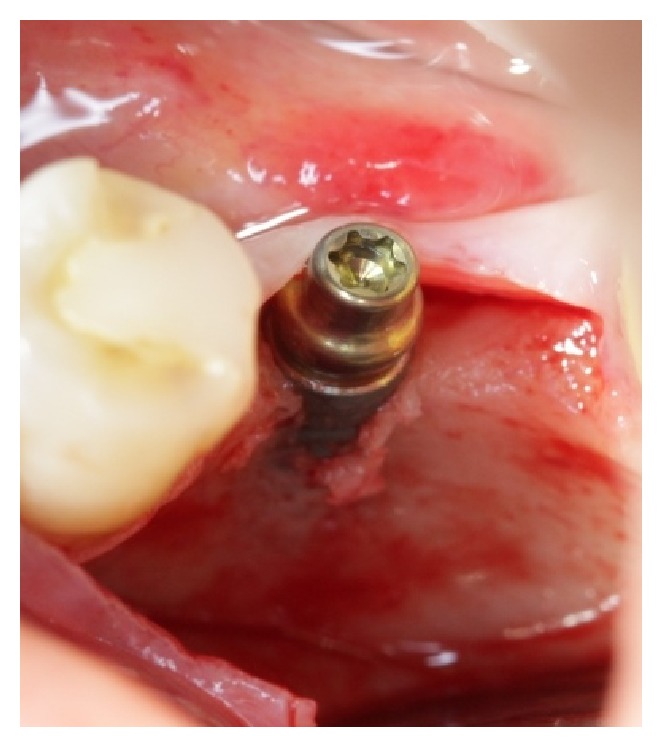
Narrow diameter implant (3,3 × 12 mm) placed on the lower left 1st molar area.

**Figure 3 fig3:**
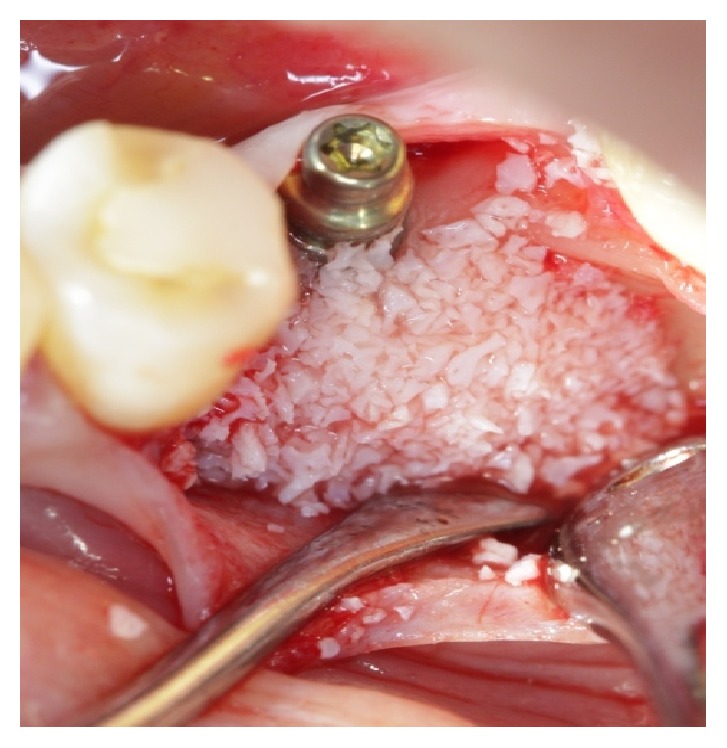
Xenograft to cover the exposed threads.

**Figure 4 fig4:**
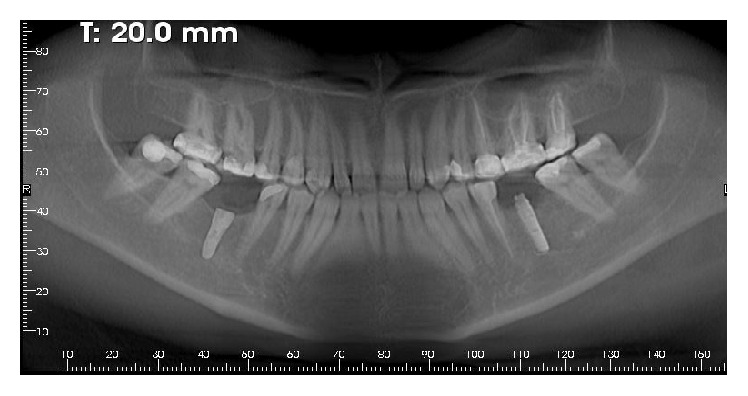
Panoramic X-ray after implant placement.

**Figure 5 fig5:**
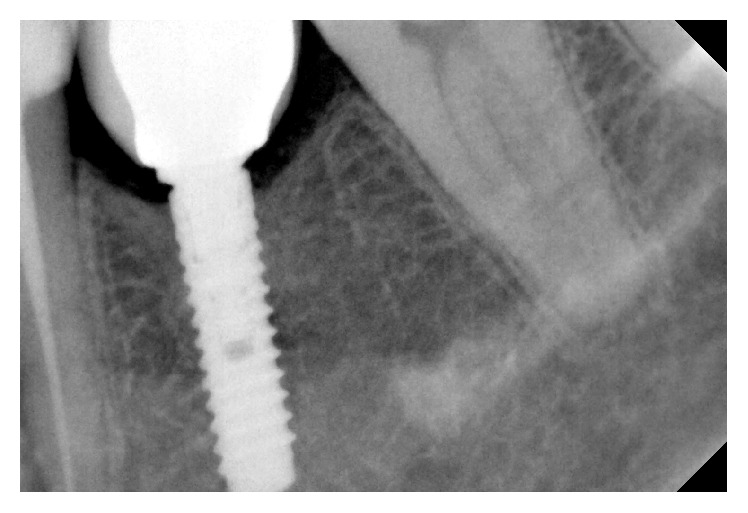
Periapical X-ray immediately after loading.

**Figure 6 fig6:**
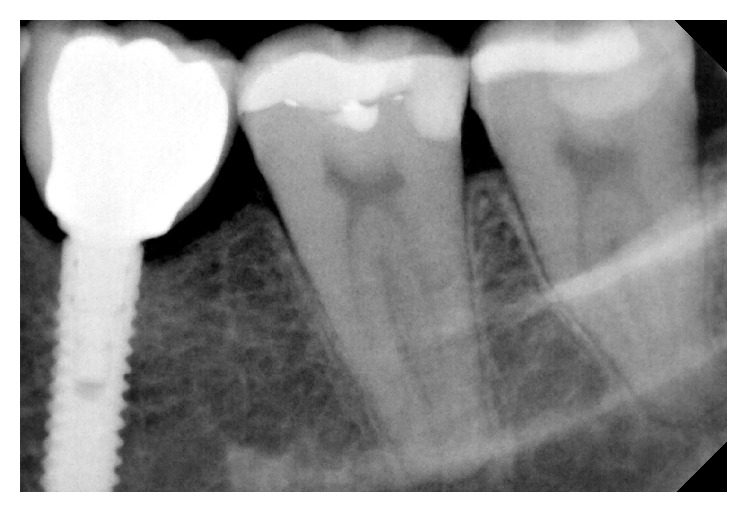
Periapical X-ray 1 year after loading.

**Figure 7:**
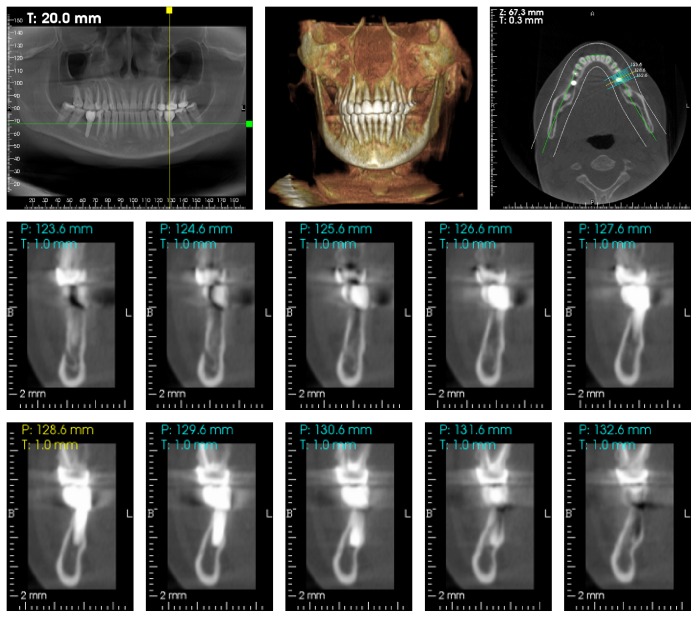
Cbct. 2 years after loading.

Clinical case 1, Figures 1 [Fig fig2] [Fig fig3] [Fig fig4] [Fig fig5] [Fig fig6]–7 show an NDI placed to replace a lower molar, with a follow-up period of 2 years

Most of the studies that evaluate the survival/success rate of NDIs focus on implants placed in the area of lower incisor and upper lateral incisor [2]. Only recently has data been published regarding the use of NDIs in the posterior jaws, thus, demonstrating an equivalent success rate to standard diameter implants [3, 4, 7, 18–20].

Splinting narrow diameter implants with wider implants or with natural abutments is one prosthetic modality reported in many studies. However, it has been observed that narrow diameter implants used alone could be a reliable treatment for posterior jaw or for full mouth rehabilitation [2].

The recent systematic review of Assaf et al. [4] demonstrated that implant therapy using NDIs in the posterior jaw is a reliable modality provided that the clinician follows certain guidelines. Implant diameter remains one of many other factors affecting implant survival, among which are implant surface and length and the osseous quality and the practitioner's learning experience curve [7].

It is therefore essential when restoring the posterior jaw to understand the complexity of the factors that enhance the durability of the treatment with small diameter implants compared to implants of standard diameter placed after bone augmentation.

This paper is an clinical study in which NDI from different implant systems was used to replace missing molars. In that sense, the paper aims at showing their reliability as an alternative option in the treatment of moderately resorbed posterior ridges when lateral bone augmentation cannot be performed. It is noteworthy that the follow-up extends from 1 to 11 years.

## 2. Materials and Methods

**Figure 8:**
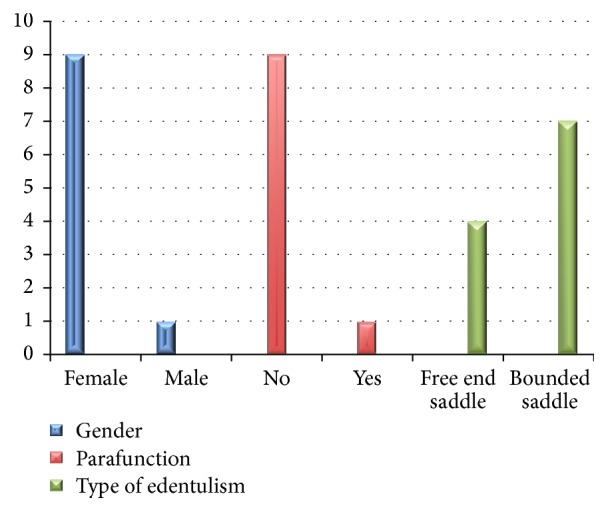
Patient's distribution according to gender, parafunction, and implant's distribution according to the type of edentulism. Nine females and 1 male. Nine patients without parafunction and 1 with parafunction. Seven out of 11 implants were in bounded saddle and 4 in free end saddle.

Between 2004 and 2012, eleven NDIs were placed for 10 patients who had moderate horizontal bone resorption at molar edentulous segments.  Figure 8 shows patients' distribution according to gender, parafunction, and implant's distribution according to the type of edentulism.

The patients' medical or/and financial statuses were also other good reasons for this treatment modality. The follow-up period ranged from 1 to 11 years. All implants were restored with fixed restorations (single crowns or fixed partial dentures). Finally, they were loaded with a conventional loading protocol.  Table 1 summarizes all evaluated parameters.

Inclusion criteria are as follows:

1. bone thickness between 5 and 7 mm,
2. vertical bone length of 12 mm above the inferior alveolar canal or 10 mm below the sinus allowing a placement of at least 10 mm height NDI,
3. NDI position in bounded molar region or free end saddle,
4. bone quality type 1, 2, or 3 according to the classification of Lekholm and Zarb [21],
5. NDI with appropriate macro- and microgeometry, that is, an external design allowing an acceptable initial stability and optimal surface preparation to enhance bone implant contact.

Minimum patient documentations included preoperative X-ray, post-operative X-ray before loading, post-operative X-ray after loading in addition to an another one 12 months later. Here, it is significant to mention that all implants were placed by one surgeon.

### 2.1. Surgical Protocol

Antibiotics (amoxicillin 500 mg TID for 5 days), analgesics, and anti-inflammatory medication (ibuprofen 600 mg TID from 3–7 days, depending on patient need) and chlorhexidine mouth rinse (TID for 7 days) were started one day prior to the surgery. Patients were treated under strict sterile conditions. Local anesthesia (articaine hydrochloridum 7200 mg/1.8 mL, adrenalin 1800 mg/1.8 mL) was provided. Full thickness flap designs/surgical protocols were released, NDIs were placed according to manufacturers' recommendations, hemostasis was achieved immediately after surgery, and postoperative instructions were given to each patient.

### 2.2. Final Prostheses Delivery

Final prosthodontics rehabilitations were carried out 2 to 6 months after implant placement, depending on bone quality and NDI initial stability. A final pick-up impression was taken using a special tray. The appropriate abutment was selected for each case. During the final prosthetic visit, the abutments were torqued to 35 Ncm using a dynamometric wrench. Metal-ceramic or metal free crowns were cemented with self-adhesive resin cement (Rely X Unicem, 3M ESPE, Seefeld, Germany). Special care was given to eliminate any gingival excess of cement material.

### 2.3. Follow-Up Visits

Patients were recalled for clinical examination visits after one month and then again after six months. After that, they were recalled once every year up to eleven years. A panoramic X-ray or an intraoral radiograph was taken to each implant site by the end of the first year. Implant success was assessed according to the criteria defined by Buser et al. [15]. In more specific terms, the implant was considered successful if the following parameters were met: (1) the absence of recurring peri-implant infection with suppuration; (2) the absence of persistent subjective complaints such as pain, the foreign body sensation, or dysesthesia; (3) the absence of a continuous radiolucency around the implant; and (4) the absence of any detectable implant mobility. These criteria have proven to be effective in defining the success of an implant system and evaluating long term results in clinical trials. By considering these outcome measures, all the implants followed in our study were judged according to their ability to satisfy the previously cited criteria, with an observed success rate of 100%.

Table 1 summarizes all evaluated parameters.

## 3. Discussion

 Narrow diameter implants are commonly used in areas where ridge dimensions are narrow or space is limited [22]. Contrary to anterior sites, the clinician's choice is restricted to only questionable ridge volume in posterior sites. Unfortunately, in this area, partial edentulism is usually long-dated and often preceded by bone loss around the teeth prior to their loss [23]. This challenging context complicates or even prohibits the placement of SDIs unless ridge augmentation is performed. Moreover, when implants are restored, they are submitted to higher levels of stress than when they would be in the anterior sites, giving a more critical role to biomechanical considerations [21]. From a pure mechanical point of view, manufacturers are prompted to improve the resistance of the implants via innovations in designs and materials. Lately, studies have shown that NDI made with titanium-zirconium alloy could be an acceptable option in compromised cases [19].

The success of NDI and the effect of diameter must be considered mainly on the long term. Javed and Romanos [16] have shown that the role of implant diameter in long term survival of dental implants is secondary. In fact, the achievement of primary stability during implant placement and the patient's postsurgical hygiene are critical factors for implant success in the posterior jaw.

Some demanding situations such as reduced ridge width and patient compromised medical status (patient tolerating only simple surgeries) are challenging to both the patient and the clinician. Hence, either the implant therapy has to be disregarded or an NDI has to become the only choice.

In case of free end saddle edentulism, NDI could be very useful since it provides the only possible fixed solution via implant-born restorations. Otherwise, a removable partial denture becomes a must with its unfavorable effect to both the residual teeth and the edentulous ridge [19].

The improvement of implant macro- and microgeometry has been another motive for the clinician to select NDI as an available treatment in the molar area. Implant initial stability and bone implant contact are critical factors for implant success, and both are related to implant macro- and microgeometry [5].

The recent systematic review of Assaf et al. [4] showed that NDI could be used in the posterior jaw under limited conditions. They proposed several surgical and prosthetic guidelines for a safe use.

In our study, 11 NDIs were placed for 10 patients, 9 females and 1 male. All were placed in type 2 or 3 bone according to the clinician's tactile evaluation. The patients had no signs or symptoms of parafunction, except for one bruxer. Five NDIs were splinted to wider-diameter implants. Two were splinted to another NDI, whereas four were restored with a single crown. Strict occlusal considerations were applied: slight contact in centric occlusion and no contact in lateral movement.

The minimum width was 3.3 mm and the minimum height was 10 mm. All implants used have optimal macro- and microgeometry.

**Figure 9:**
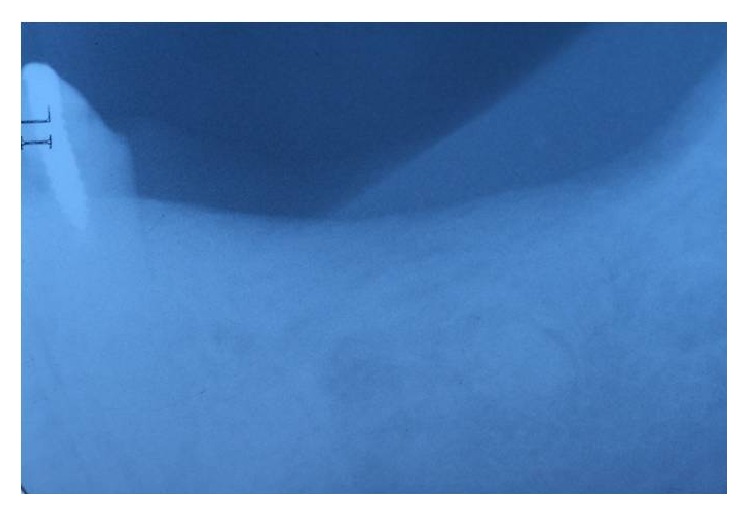
Periapical X-ray showing teeth loss on the lower left second premolar, first and second molar.

**Figure 10 fig10:**
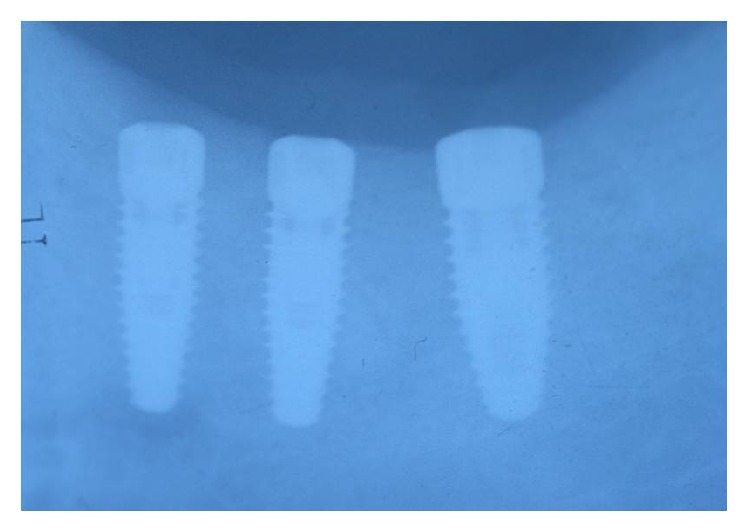
Three implants to replace the missing teeth, the implants placed on the second premolar and first molar are narrow diameter implant (3,5 mm).

**Figure 11 fig11:**
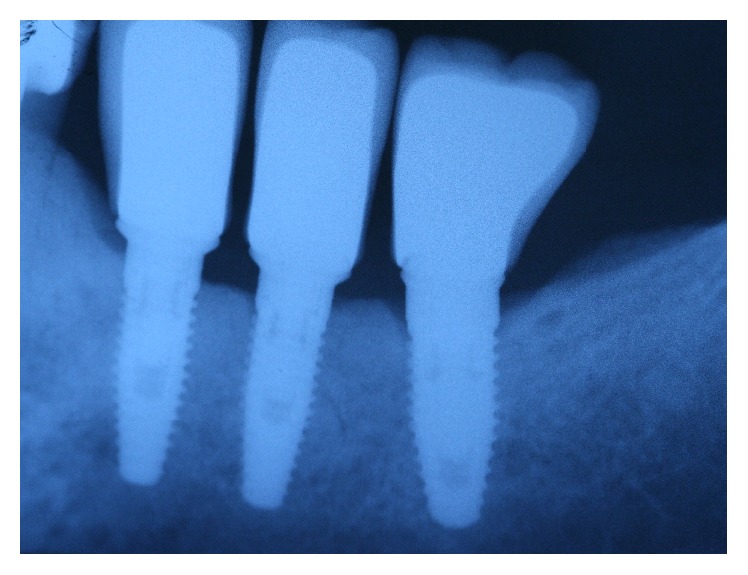
Periapical X-ray 6 months after crown cementation.

**Figure 12 fig12:**
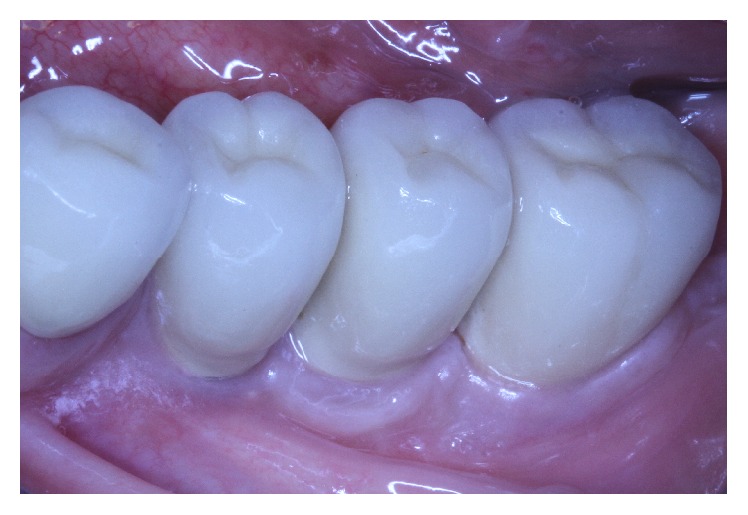
Clinical situation 7 years after loading.

**Figure 13 fig13:**
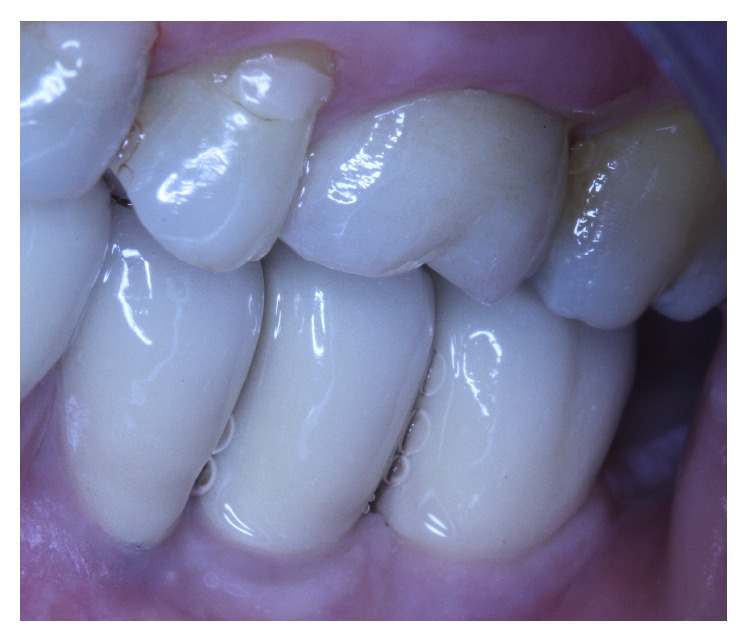
Centric occlusion.

**Figure 14 fig14:**
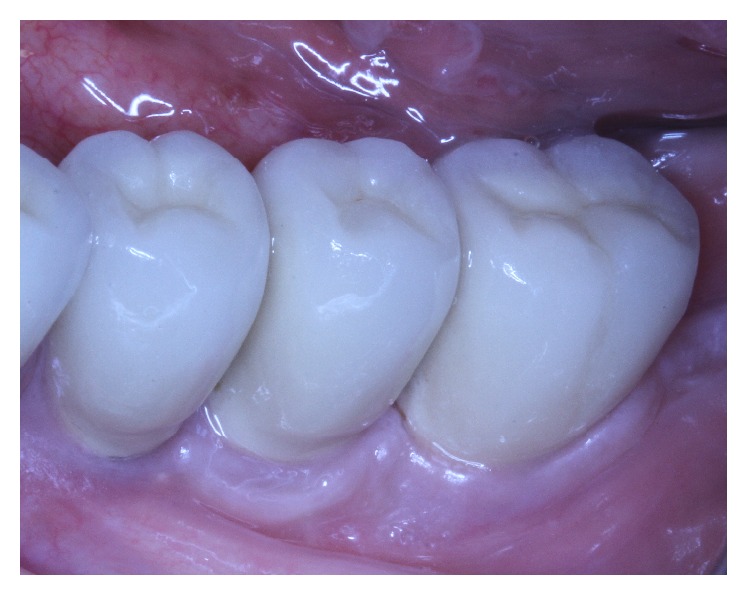
Clinical situation 11 years after loading.

**Figure 15:**
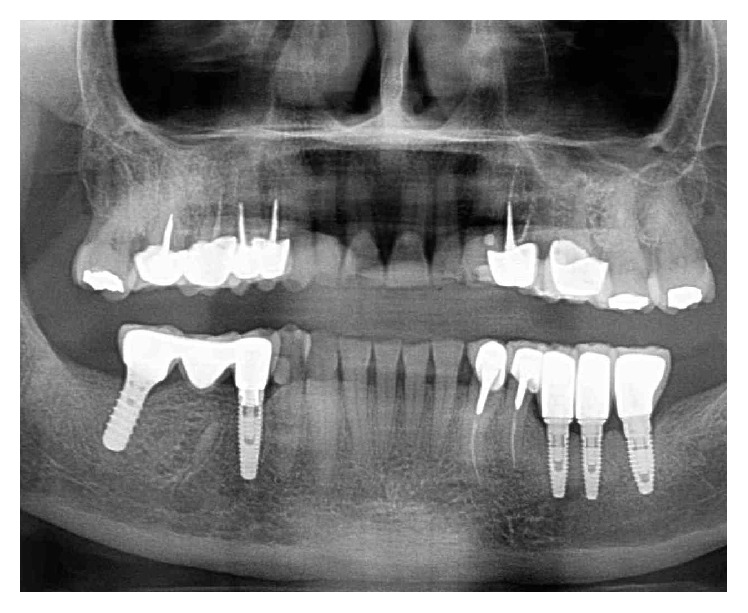
Panoramic X-ray 11 years after.

Surprisingly, the only NDI which was placed to the bruxing patient showed optimal peri-implant bone level and probing depth after 11 years of functioning. This implant was placed in bounded saddle adjacent to two standard diameter implants, all restored with single crowns (see clinical case 2, Figures 9 [Fig fig10] [Fig fig11] [Fig fig12] [Fig fig13] [Fig fig14]–15).

## 4. Conclusion

In case of moderate horizontal bone resorption, NDI may be a reliable option to replace a molar, if the following conditions are satisfied:

1. bone quality type 1, 2, or 3,
2. minimum implant length of 10 mm,
3. implant protective occlusion,
4. patient with no history of parafunction,
5. implant with appropriate macro- and microgeometry,
6. bone thickness between 5 and 6 mm.

Further observational and randomized controlled studies could provide deeper evidence-based conclusions concerning the use of NDI in the posterior jaws.

## Figures and Tables

**Table 1:** The different parameters evaluated in our case series. Patient gender (F, female; M, male ), NDI position in the arch, bone management, type of prosthesis (fixed partial denture = FPD or single crown (SC)), loading protocol, type of edentulism (F = free end saddle, B = bounded saddle), splinted to wider diameter implant, nature of opposing arch, presence of a cantilever, follow-up period, and median probing depth.

Patient name, gender, age	NDI position	NDI diameter length micro- and macrogeometry	Parafunction	Bone management	Type of prosthesis	Loading protocol	Free end (F) or bounded (B) saddle	Splinted to wider diameter implant	Nature of opposing arch	Presence of a cantilever	Follow-up period	Mean probing depth
Patient 1: F, 55 years	46	3,3 × 10 Straumann SP cylindrical shape, monothread, SLA surface	No	No	FPD	3 months	F	No	Fixed *metal*-ceramic	Yes (median)	4 years	3 mm

Patient 2: F, 49 years	36, 47	Nucleoss (2) 3,4 × 10 conical shapes, Maxicell surface (SLA)	No	No	FPD	4 months	36: B. 47: F	Yes	Fixed *metal*-ceramic	No	3 years	4 mm

Patient 3: F, 44 years	36	3,4 × 12 Nucleoss conical shapes, Maxicell surface (SLA)	No	No	FPD	3 months	F	Yes	Fixed *metal*-ceramic	No	4 years	3 mm

Patient 4: F, 53 years	46	3,5 × 11,5 Astra Tech OsseoSpeed, TiO_2_ blasted surface	No	No	FPD	4 months	F	Yes	Fixed *metal*-ceramic	Yes (median and distal)	9 years	5 mm

Patient 5: F, 64 years	46	3,4 × 10 Nucleoss conical shapes, Maxicell surface (SLA)	No	No	Full-arch fixed	2 months	B	Yes	Removable denture	Yes (median)	2 years	3 mm

Patient 6: F, 39 years	36	3,4 × 10 Nucleoss conical shapes, Maxicell surface (SLA)	No	No	FPD	3 months	B	Yes	Fixed *metal*-ceramic	No	5 years	3 mm

Patient 7: M, 57 years	46	3,3 × 12 Straumann SP cylindrical shape, monothread, SLA surface	No	No	FPD	3 months	B	No	Fixed *metal*-ceramic	No	2 years	3 mm

Patient 8: F, 51 years	36	3,5 × 11,5 replace select conical shape, TiUnite surface	Yes	No	FPD	4 months	B	No	Natural teeth	No	11 years	3 mm

Patient 9: F, 49 years	36	3,3 × 12 Straumann BL cylindrical shape, monothread, SLA surface	No	Yes (xenograft)	SC	6 months	B	No	Fixed *metal*-ceramic	No	1 year	3 mm

Patient 10: F, 62 years	46	3,5 × 12 Biohorizon cylindrical shape, Laser-Lok surface	No	Yes (allograft)	SC	4 months	B	No	Fixed *metal*-ceramic	No	1 year	3 mm
